# “Natural Regulators”: NK Cells as Modulators of T Cell Immunity

**DOI:** 10.3389/fimmu.2016.00235

**Published:** 2016-06-14

**Authors:** Iona S. Schuster, Jerome D. Coudert, Christopher E. Andoniou, Mariapia A. Degli-Esposti

**Affiliations:** ^1^Immunology and Virology Program, Centre for Ophthalmology and Visual Science, The University of Western Australia, Perth, WA, Australia; ^2^Centre for Experimental Immunology, Lions Eye Institute, Nedlands, WA, Australia

**Keywords:** NK cells, immune regulation, adaptive immunity, viral infection, ILC

## Abstract

Natural killer (NK) cells are known as frontline responders capable of rapidly mediating a response upon encountering transformed or infected cells. Recent findings indicate that NK cells, in addition to acting as innate effectors, can also regulate adaptive immune responses. Here, we review recent studies on the immunoregulatory function of NK cells with a specific focus on their ability to affect the generation of early, as well as long-term antiviral T cell responses, and their role in modulating immune pathology and disease. In addition, we summarize the current knowledge of the factors governing regulatory NK cell responses and discuss origin, tissue specificity, and open questions about the classification of regulatory NK cells as classical NK cells versus group 1 innate lymphoid cells.

## Introduction

Natural killer (NK) cells were identified in the 1970s as natural effector cells with the capacity to lyse tumor cells (1, 2). The role of NK cells as effectors against transformed and virally infected cells has been well established (3). Regulated through a range of activating and inhibitory receptors (4), NK cell activation results in cytotoxic degranulation and the production of inflammatory cytokines, such as interferon-γ (IFN-γ) and tumor necrosis factor-α (TNF-α) (5). While the importance of NK cells as frontline responders remains undisputed, regulatory functions of NK cells are being increasingly appreciated in several settings and especially in the regulation of immune responses during viral infections (6–12). A regulatory role of NK cells is also emerging in the context of reproduction [reviewed in Ref. (13)]. A major issue that is still to be resolved is the nature of these “natural immune regulators,” and whether they represent classical NK cells or a population of the recently defined innate lymphoid cells (ILCs).

## Impact through Cytokine and Chemokine Production

Activated NK cells can express a number of secreted molecules that may augment or dampen immune responses. NK cell-derived IFN-γ and TNF-α have antiviral effects and play a role in the activation and/or maturation of dendritic cells (DCs) (14–16), macrophages, and T cells (5, 17). Moreover, NK cells can produce granulocyte-macrophage colony-stimulating factor (18), IL-5, IL-13 (19), and IL-10 (6), thereby impacting various downstream immune responses. NK cells can also affect immune responses through the production of chemokines, such as CCL3, CCL4, and CCL5, which recruit other effector cells to sites of inflammation (20).

## Impact on DCs and the Priming of Adaptive Immune Responses

In addition to cytokine- and chemokine-mediated effects, NK cells can affect the priming of adaptive immune responses through killing of infected or transformed cells (7, 16, 21, 22). Early elimination of murine cytomegalovirus (MCMV)-infected cells through recognition of the viral protein m157 by Ly49H^+^ NK cells has been associated with decreased levels of type I IFN production by plasmacytoid DCs (21). As a result, DCs were maintained in higher numbers, augmenting the very early priming of antiviral CD8^+^ T cell responses. However, this was a transient effect, as Ly49H^+^ NK cells also eliminate MCMV-infected DCs, ultimately reducing T cell priming and thereby curbing ongoing T cell responses (7).

Similarly, early depletion of NK cells in a mouse model of lymphocytic choriomeningitis virus (LCMV) infection improved DC priming of virus-specific T cells (11). Together, these studies establish a role for NK cells in the regulation of DCs and, as a consequence, the priming of adaptive T cell responses.

## NK Cells as Direct Modulators of Adaptive T Cell-Mediated Immunity

As mentioned above, NK cells can contribute to the activation of T cells through the production of cytokines that promote T cell priming and differentiation (5). In addition, NK cells can express costimulatory molecules, such as OX40 ligand, allowing them to directly promote T cell proliferation (23). Direct NK cell-mediated inhibition of T cell responses has also been reported. In this regard, a number of different *in vitro* and *in vivo* studies in human and mouse models have shown that NK cells are capable of directly killing T cells. An *in vitro* approach studying the role of human NK cells in the response to intracellular antigen revealed that activated NK cells can eliminate regulatory T cells (24). Direct elimination of effector CD4^+^ T cells by NK cells has been reported in mouse models of chronic inflammation (25) and graft-versus-host disease (26). Similarly, human NK cells have been reported capable of killing activated T cells (27, 28). *In vitro* experiments investigating the sensitivity of T cells to NK cell-mediated killing revealed that, while resting T cells were resistant to lysis by NK cells, recently primed T cells were susceptible (29). Susceptibility to killing was limited to the first few days following T cell activation, and declined thereafter, with T cells regaining sensitivity to NK cell killing upon re-encountering cognate antigen. The sensitivity of activated T cells was associated with the upregulation of ligands for the NK cell activating receptor NKG2D. The role of NKG2D-mediated activities in the killing of T cells by NK cells *in vivo* is more complicated, with reports that NKG2D is both important (8, 9) or irrelevant (10, 30) being available. Furthermore, blocking inhibitory signals delivered upon engagement of the non-classical MHC I molecule Qa-1 has been shown to allow the NK cell-mediated elimination of autoreactive T cells, resulting in reduced pathology in a model of experimental autoimmune encephalomyelitis (31). Although published studies mostly report NK cell-mediated effects on CD4^+^ T cells, suggesting that CD4^+^ T cells may be more susceptible to NK cell-mediated elimination, both CD4^+^ and CD8^+^ T cells can be eliminated by NK cells, and this primarily involves perforin-dependent activities (8–10, 32). Elimination of T cells involving the TNF-related apoptosis-inducing ligand (TRAIL) (12, 27, 33) and Fas (26) has also been reported.

## NK Cells in the Regulation of Antiviral T Cell Responses

Even though the interactions between NK cells and T cells have been studied in a number of different models, the most extensive investigations have been undertaken in the setting of viral infections. In this context, it is increasingly evident that, in addition to contributing to the elimination of infected cells, NK cells can also limit antiviral immunity (7, 9–12). Specifically, NK cells have been shown to impair both initial and ongoing antiviral T cell responses.

### NK Cells as Regulators of Early Antiviral T Cell Immunity

A frequently used model to investigate the role of NK cells in viral infection is MCMV. The first indication that NK cells could modulate T cell responses came from studies showing that their depletion in C57BL/6 mice at the time of MCMV infection resulted in increased T cell proliferation and higher numbers of T cells expressing IFN-γ (34). This important finding was extended by our studies showing that NK cells limited the function and longevity of antiviral T cell responses *via* the elimination of MCMV-infected DCs (7). Notably, the more robust antiviral T cell activities generated in the absence of early NK cell responses expedited the control of chronic MCMV infection (7). Furthermore, it has been reported that a mutation in the gene encoding the NK cell activating receptor NKp46, which leads to hyperreactivity of NK cells, results in suboptimal anti-MCMV T cell responses (35). Most recently, our studies have revealed an immune-regulatory function for NK cells in the salivary glands, a site of chronic MCMV infection (12). Viral control in the salivary glands is directly dependent on CD4^+^ T cells, with more robust antiviral CD4^+^ T cell responses resulting in accelerated viral control. Notably, CD4^+^ T cells accumulating in the salivary glands during chronic MCMV infection upregulate the receptor for TRAIL, rendering them susceptible to TRAIL-mediated activities. These activities were found to be mediated by TRAIL on NK cells (12), with timed NK cell depletion or deficiency in TRAIL resulting in elevated numbers of antiviral CD4^+^ T cells and enhanced viral control. The specific upregulation of NKG2D ligands on CD4^+^, but not CD8^+^ T cells, and the NKG2D expression on TRAIL^+^ NK cells provided strong support for an important role being mediated by these additional interactions in determining the fate of TRAIL-R^+^ CD4^+^ T cells upon encounter with TRAIL^+^ NK cells.

Similar to observations made for MCMV infection, there is accumulating evidence supporting a role for NK cells in the regulation of antiviral T cell responses to LCMV. A study investigating the role of the NK cell receptor 2B4, revealed that 2B4-deficient mice had reduced numbers of activated CD8^+^ T cells and consequently prolonged viral persistence (32). This phenotype could be reverted by depletion of NK cells, indicating that the absence of 2B4 leads to NK cell-mediated elimination of LCMV-specific CD8^+^ T cells.

Further studies have supported the hypothesis that NK cells regulate T cell responses during both an acute and chronic LCMV infection. Following both types of LCMV infection, NK cells showed enhanced cytotoxicity and were capable of eliminating T cells that had been activated *in vitro* (9). Notably, in the absence of NK cells, LCMV infection resulted in increased frequencies of LCMV-specific CD8^+^ T cells, significantly reduced viral titers, and less virus-induced hepatitis, suggesting that NK cells promote the development of chronic infection (9). Protection against T cell exhaustion was also reported in the absence of NK cells (11). A concurrent study confirmed the regulatory effect of NK cells on the generation of antiviral T cell responses, immune pathology, and viral control (10). Here, however, the authors extended previous findings and reported that viral dose had a significant impact on how NK cells affected antiviral immune responses. The varying effects of NK cells in infections with different LCMV viral doses were due to changes in the patterns of T cell-mediated pathology. In mice infected with a high viral dose, NK cells prevented mortality but promoted viral persistence. Here, NK cell depletion resulted in elevated T cell numbers, reduced T cell exhaustion, but increased mortality, indicating a protective effect of NK cells in this setting. In contrast, the severe immune pathology associated with infection with a medium LCMV viral dose was abrogated by depletion of NK cells, and complete viral clearance was achieved with 100% survival. Although ultimately NK cells suppressed CD8^+^ T cell responses, the authors propose that this is mediated indirectly *via* the elimination of CD4^+^ T cells (10).

Together, these studies clearly outline a role for NK cells in the regulation of primary T cell responses to viral infection and open the possibility that the regulatory effects of NK cells may also modulate long-term antiviral responses and the generation of memory.

### Regulatory NK Affect Ongoing Antiviral T Cell Immunity and Memory Generation

In MCMV infection, mice lacking the capacity to eliminate MCMV-infected DCs through Ly49H^+^ NK cells generated more robust T cell responses, with significant differences in both kinetics and functionality (7). Importantly, these differences are maintained in the long term, with stronger cytokine and chemokine expression and increased retention of leukocytes in tissues observed in mice lacking early Ly49H-mediated NK cell activities (unpublished observations, S. V. VanDommelen, Iona S. Schuster, Christopher E. Andoniou, Mariapia A. Degli-Esposti).

An effect on ongoing antiviral responses was also noted in MCMV-infected mice lacking TRAIL^+^ NK cells in the salivary glands (12). The lack of TRAIL^+^ NK cells resulted in increased accumulation of antiviral T cells and improved viral control in the salivary glands. However, the failure to restrain CD4^+^ T cell responses was associated with an increase in autoantibody production, the formation of tertiary lymphoid-like structures, and, most importantly, the loss of tissue function in the affected exocrine glands indicating the development of an autoimmune disease with the characteristics of human Sjogren’s syndrome (12). These observations are of particular interest as this study provided the first evidence for a physiological effect of NK cell immune regulation, with NK cells playing a crucial role in maintaining a balance between protective immunity and excessive pathologic or autoreactive immune responses.

Similar to MCMV infection, the immunomodulatory functions of NK cells in LCMV infected mice not only affect early antiviral immune responses but also modulate ongoing antiviral immunity and the generation of immunological memory (11, 30, 36). First indicators of the long-term effects of NK cells on anti-LCMV T cell responses were the reduced frequencies of virus-specific T cells in mice lacking the NK cell inhibitory receptor 2B4 observed up to 100 days post-LCMV infection (32), and the observation that T cell exhaustion, as defined by PD-1 and Lag-3 expression, was decreased in NK-cell depleted mice (11). Further studies demonstrated that NK cell depletion in the initial stages of acute LCMV infection, as well as Pichinde virus infection, specifically altered the generation of memory precursor effector cells and resulted in T cell memory with improved functional capacity (30). Furthermore, NK cell-depleted mice had elevated numbers of T follicular helper cells (Tfh) and germinal center B cells, resulting in more sustained antibody responses (30). The latter results were corroborated by data demonstrating that mice depleted of NK cells just before LCMV infection generated a more robust Tfh response facilitating greater germinal center B cell and plasmablast formation, elevated concentration of anti-LCMV antibodies, and improved control of chronic infection (36).

**Figure 1:**
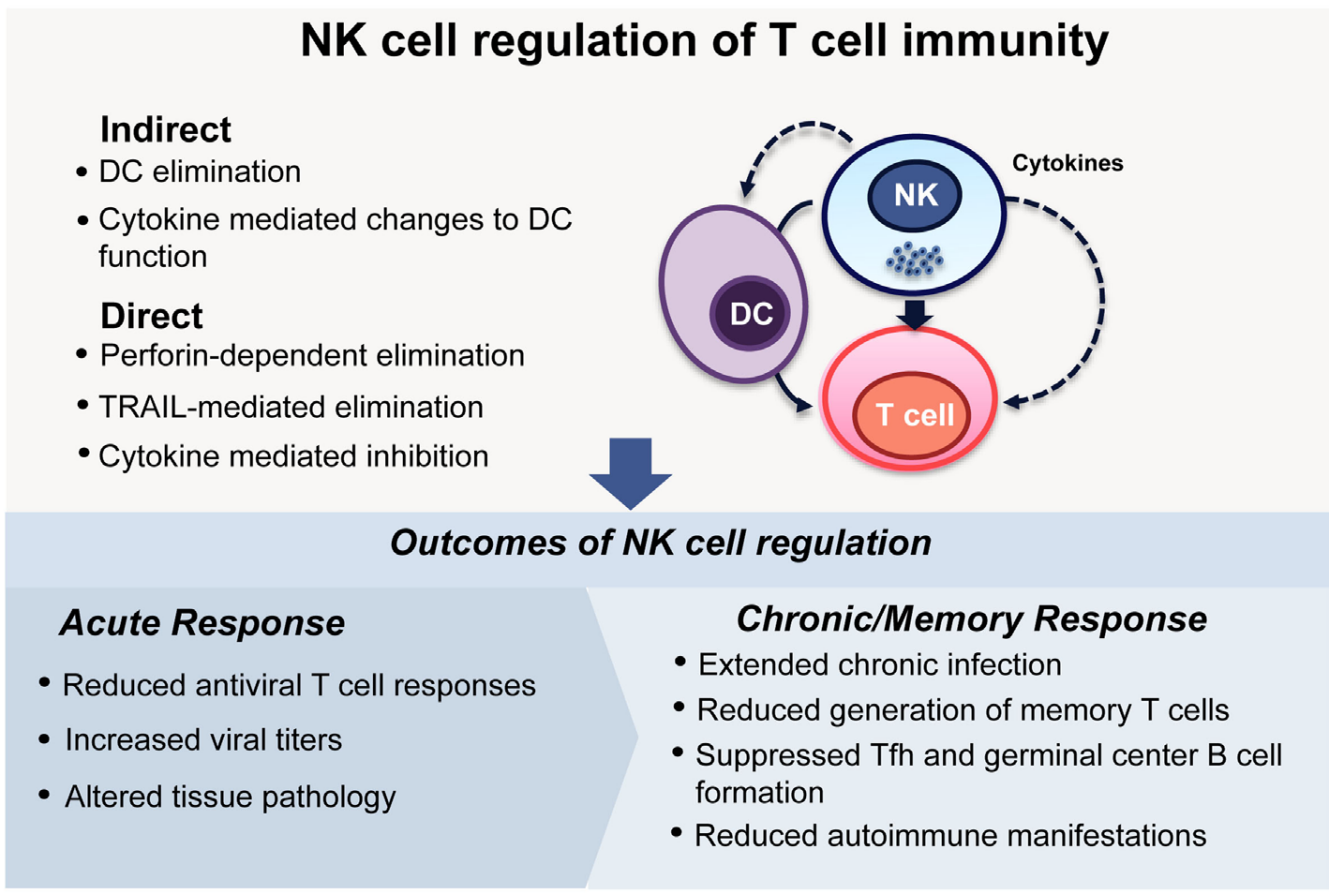
**NK cell regulation of T cell immunity**. NK cells can modulate the activities of T cells either indirectly, by eliminating, or affecting the functions of antigen-presenting cells, especially DCs, or through direct interactions with T cells themselves. During viral infections, these regulatory interactions can lead to differing outcomes depending on the infecting viral pathogen and the phase of the infection. During acute infection, NK cell regulation can dampen antiviral T cell responses (7, 9–11), lead to increased viral loads (9–11), and ultimately determine the extent of pathology in tissues (9, 10). In infections that have a chronic phase, NK cells can determine the longevity of the infection and indeed extend it (7, 11, 12, 33, 36). Memory T cell responses are generated at reduced frequencies (30), and antibody responses can be compromised due to impaired formation of germinal centers (30, 36). This apparently incongruous dampening of immune response is reconciled by the finding that curbing chronic immune responses following viral infections is critical to reducing the development of autoimmunity (12).

Overall, these studies form a strong body of evidence supporting the hypothesis that NK cells not only affect the generation of primary T cell responses to viral pathogens but also alter the quantity and quality of ongoing responses, including T and B cell memory responses and long-term protection. Although the NK cell-mediated moderation of both primary antiviral T cell responses and the ensuing generation of memory may at face value appear detrimental to the host, the development of autoimmunity observed in mice lacking regulatory NK cell functions indicates that, in physiological settings, NK cells contribute to the balancing of immune responses (Figure 1).

Much remains to be understood about the immune-regulatory role of NK cells, including their impact on adaptive immunity at late stages of persistent viral infections. Interestingly, in a model of Friend retrovirus infection, NK cell depletion in the acute phase of infection impaired viral control, but depletion of NK cells during chronic infection led to increased numbers of activated CD8^+^ T cells and expedited viral control (37). A study by Peppa et al. (33) outlined a role for NK cells in limiting hepatitis B virus (HBV)-specific CD8^+^ T cell numbers. Analysis of PBMCs from chronically infected patients cultured in the presence or absence of NK cells showed increased numbers of activated CD8^+^ T cells in NK cell-depleted cultures. A significant upregulation of TRAIL receptor 2 (TRAIL-R2) was noted on hepatic CD8^+^ T cells from chronically HBV-infected patients, compared with healthy controls, and blocking TRAIL augmented the survival of hepatic CD8^+^ T cells cultured with PBMCs (33). These results indicate that the immune-regulatory functions of NK cells are not only restricted to mouse models of infection but are also observed in the human system. Whether NK cells play similar immunoregulatory activities in other settings of chronic disease remains to be described.

## Regulating the Regulators

Factors that modulate the regulatory functions of NK cells in inflammatory settings have recently been identified. NK cells from mice with a mutation in the gene for the NK cell activating receptor NKp46 failed to downregulate the transcription factor *Helios*, resulting in overly responsive NK cells, which in turn weakened T cell responses in inflammatory settings, such as viral infection (35). Other factors regulating the susceptibility of T cells to NK cell-mediated elimination include the transcription regulator NOD-like receptor caspase recruitment domain containing protein 5 (NLRC5) (38). The absence of NLRC5 in activated T cells responding to different inflammatory stimuli resulted in significantly reduced MHC I expression and rendered these cells susceptible to NK cell killing. The susceptibility of T cells to NK cell killing can also be affected by type I IFN, and *Ifnar1*^−/−^ T cells were found to have reduced expression of the NK cell inhibitory ligands MHC I and Qa-1b (39) and elevated expression of ligands for the NK cell activating receptor NKp46 (40), leading to increased NK cell killing of LCMV-specific T cells.

## Regulatory NK Cells: ILC1 Versus Classical NK Cells

Even though immune-regulatory effects of NK cells are slowly being unraveled, many questions remain unanswered regarding the generation, phenotype, and tissue specificity of NK cells with immune-regulatory capacity. Addressing these questions has been complicated by the recent identification of novel populations of ILCs. NK cells have been classified as being a subclass of group 1 ILCs (41). The group 1 ILCs are characterized by their expression of the T-box transcription factor T-bet, an ability to produce IFN-γ, and an inability to produce T_H_2- and T_H_17-associated cytokines (41). The group 1 ILC can be subdivided into NK cells and ILC1, but the distinction between the subsets is hardly straightforward and appears to be tissue and activation dependent (42). In liver, at steady state, for example, ILC1 can be distinguished from NK cells by the expression of CD49a and TRAIL and a lack of CD49b expression and the transcription factor Eomesodermin (Eomes) (43, 44). NK cells are strongly cytotoxic and express high levels of perforin and granzymes, whereas ILC1 express low levels of perforin, but show increased expression of cytokines, including IFN-γ and especially TNF-α (43, 45). Despite reduced perforin expression, hepatic TRAIL^+^ NK cells were reported to kill tumor cells in both a TRAIL-dependent and -independent manner (46) and appear to contribute to liver pathology in chronic HBV sufferers by eliminating hepatocytes (47, 48). Together, these data suggest that hepatic ILC1 cells may have a greater cytotoxic potential than currently assumed.

In addition to being found in the liver, ILC1 cells have been described in other tissues, including the gut, skin, uterus, and salivary glands (45, 49). Differentiating between NK cells and ILC1 is of particular interest in the salivary glands, where TRAIL^+^ NK cells limit anti-MCMV CD4^+^ T cell responses (12). Tessmer et al. (50) described steady-state salivary gland NK cells as a phenotypically distinct population, based on their integrin and activation marker profile, with these cells unable to mount efficient cytotoxic responses to various stimuli. Following MCMV infection, a lack of NK cell recruitment was noted, suggesting that the NK cell regulatory response in the salivary glands is mediated by proliferation of tissue-resident cells. This characteristic of salivary gland NK cells is shared with ILC1 cells that are thought to be primarily tissue resident (45). These results differ from our own, where regulatory TRAIL^+^ NK cells seem to be recruited into the salivary glands in response to MCMV infection (12). Possible explanations for this disparity include differences in the timing of NK cell transfer, the strain of mouse, and/or the strain of MCMV used in the different experiments.

The ILC populations found in the salivary glands have phenotypic profiles consistent with both NK cells and ILC1 (49). Interestingly, the development of these cells under homeostatic conditions was found to be independent of the transcription factor Nfil3, suggesting that NK cells present in the salivary glands at steady state are ILC1 (49, 51). However, salivary gland NK cells express both T-bet and Eomes, consistent with an NK cell phenotype. Furthermore, under inflammatory conditions, such as viral infection, it appears that NK cells can be recruited to the salivary glands, and their phenotype does not conform to that of ILC1 (12). Thus, the regulatory NK cell population that balances protective antiviral immunity with potentially pathologic autoreactive immune responses appears to be classical NK cells. However, in their entirety, the available data suggest that a distinction between classical NK cells versus ILC1s may be very difficult especially in inflamed tissues, where cytokines can drastically alter the phenotype of these populations.

## Conclusion

The collective data reviewed here establish that NK cells regulate adaptive responses both during priming and at later stages. Importantly, the regulatory functions of NK cells are not limited to interactions in lymphoid tissues but have been observed in non-lymphoid organs. An intriguing question yet to be answered is whether the regulation of adaptive immune responses in tissues is mediated by ILC1, NK cells or both subsets, and if there is any plasticity between these cell populations.

## Author Contributions

All authors listed, have made substantial, direct and intellectual contribution to the work, and approved it for publication.

## Conflict of Interest Statement

The authors declare that the research was conducted in the absence of any commercial or financial relationships that could be construed as a potential conflict of interest.
